# Effects of ocean acidification and hydrodynamic conditions on carbon metabolism and dissolved organic carbon (DOC) fluxes in seagrass populations

**DOI:** 10.1371/journal.pone.0192402

**Published:** 2018-02-08

**Authors:** Luis G. Egea, Rocío Jiménez-Ramos, Ignacio Hernández, Tjeerd J. Bouma, Fernando G. Brun

**Affiliations:** 1 Department of Biology, Faculty of Marine and Environmental Sciences, University of Cadiz, Puerto Real, Cádiz, Spain; 2 Royal Netherlands Institute for Sea Research (NIOZ), Department of Estuarine and Delta Systems, Royal Netherlands Institute for Sea Research (NIOZ), Yerseke, The Netherlands; National Taiwan Ocean University, TAIWAN

## Abstract

Global change has been acknowledged as one of the main threats to the biosphere and its provision of ecosystem services, especially in marine ecosystems. Seagrasses play a critical ecological role in coastal ecosystems, but their responses to ocean acidification (OA) and climate change are not well understood. There have been previous studies focused on the effects of OA, but the outcome of interactions with co-factors predicted to alter during climate change still needs to be addressed. For example, the impact of higher CO_2_ and different hydrodynamic regimes on seagrass performance remains unknown. We studied the effects of OA under different current velocities on productivity of the seagrass *Zostera noltei*, using changes in dissolved oxygen as a proxy for the seagrass carbon metabolism, and release of dissolved organic carbon (DOC) in a four-week experiment using an open-water outdoor mesocosm. Under current pH conditions, increasing current velocity had a positive effect on productivity, but this depended on shoot density. However, this positive effect of current velocity disappeared under OA conditions. OA conditions led to a significant increase in gross production rate and respiration, suggesting that *Z*. *noltei* is carbon-limited under the current inorganic carbon concentration of seawater. In addition, an increase in non-structural carbohydrates was found, which may lead to better growing conditions and higher resilience in seagrasses subjected to environmental stress. Regarding DOC flux, a direct and positive relationship was found between current velocity and DOC release, both under current pH and OA conditions. We conclude that OA and high current velocity may lead to favourable growth scenarios for *Z*. *noltei* populations, increasing their productivity, non-structural carbohydrate concentrations and DOC release. Our results add new dimensions to predictions on how seagrass ecosystems will respond to climate change, with important implications for the resilience and conservation of these threatened ecosystems.

## Introduction

Over the last century, humans have produced large amounts of CO_2_ through activities such as fossil fuel burning, intensive agriculture and deforestation [1]. The concentration of CO_2_ in the atmosphere has risen from 280 ppm in preindustrial times to 400 ppm in 2015, as measured at Mauna Loa Observatory, which holds one of the longest records for direct measurement of atmospheric carbon dioxide [2]. By the middle of this century, atmospheric CO_2_ levels could reach more than 500 ppm and surpass 800 ppm by the end of the century [3]. During the industrial era, the ocean has absorbed about one-quarter of this anthropogenic CO_2_ [4,5], acting as a carbon sink and thus contributing to the key ecosystem service of climate regulation. A portion of the CO_2_ absorbed by oceans is stored in living biomass and sequestered in sediments but a large amount remains in its inorganic form. This increase of inorganic carbon stored in the oceans has driven a reduction in seawater pH and promoted changes in the seawater chemistry in a process commonly referred to as “ocean acidification” (OA) [6–10]. The global ocean pH is expected to fall to between 8.05 and 7.6 by the end of this century (IPCC, 2013; [11]). This raises concern about the possible impacts of these changes on marine organisms. OA is a ubiquitous stressor, which is predicted to lead to negative consequences in the future for marine organisms [12], ecosystems [13] and the provision of ecosystem services [14]. In recent years, studies have underscored the crucial role of shallow coastal ecosystems, which function not only as transition zones between land and ocean but also as filters for carbon sequestration [15,16]

Seagrasses are marine flowering plants that form one of the richest coastal ecosystems [17], providing a large number of ecological services [16–18] including long-term carbon catchment [16,18]. They cover less than 0.2% of the ocean surface but make a disproportionately high contribution to marine net primary productivity (NPP) (1%) and are responsible for approximately 15% of the carbon stored in oceans [19]. Seagrass-dominated ecosystems are highly productive habitats [20] that play an important role in the carbon cycle of coastal areas [20,21]. The excess organic matter that they produce can be exported to adjacent ecosystems in particulate or dissolved forms [22,23]. Dissolved organic carbon (DOC), with about 700 PgC, represents only 2% of the carbon pool in the ocean [24]. However, it is a central factor in the global carbon cycle [25,26], acting as a vector for quick transfer of C and energy in food webs, as it is easily assimilated by marine organisms and fully involved in the C exchange between communities [25,27]. The global net DOC exported from seagrass meadows, as calculated by Barrón et al [28], represents as much as the 46% of the global NCP of seagrass meadows, as reported by Duarte et al [29]. Thus, this released DOC may be critical in maintaining the high annual productivity of communities dominated by seagrasses [30]. However, few studies have focused on the relation between productivity and DOC flux, especially *in situ* (for review, see Barrón et al [28]). While there has been extensive research in the DOC dynamics of the open ocean (for reviews, see Hansell & Carlson [25,31]), the role of coastal ecosystems in the global DOC cycle is still inadequately understood, despite being a strong sink for ocean DOC [32].

Increased anthropogenic pressure has led to widespread loss of seagrasses in shallow estuarine and coastal zones [33]. With the decline in the global surface area of meadows measured at 7% yr^-1^ [34], seagrass meadows are the most quickly declining ecosystem on the planet. OA could either ameliorate or aggravate this current decline in seagrasses [10]. Thus, the response of seagrasses to OA must be considered for effective management of coastal regions in the future. The effects of increased CO_2_ concentrations on seagrasses may depend on the degree of carbon limitation in natural systems. Carbon limitation has been partly attributed to the thickness of the diffusive boundary layer (DBL) surrounding leaf surfaces at low current velocities [35–37] or to a relatively inefficient HCO_3_^-^ uptake system [38]. Increases in water current velocity are linked to a thinner DBL [39] and consequently, faster transfer of CO_2_ molecules from the water column to seagrass cells. As higher current velocity leads to a reduction in the DBL thickness, this favours CO_2_ uptake and photosynthesis [40]. However, increasing flow velocity may reduce photosynthetic rates due to enhanced sediment resuspension (i.e. decreasing light levels; [41]) and increased self-shading by leaves, because leaves tend to collapse onto each other when exposed to strong currents [40]. Hence, current velocity conditions can result in a net positive or negative effect on seagrass productivity, depending on which effect is stronger: an increase in CO_2_ uptake as a result of the decrease in DBL or a reduction in photosynthesis as a consequence of sediment resuspension or leaf self-shading. It is important to highlight here that hydrodynamic conditions in coastal areas worldwide may also change as a consequence of anthropogenic engineering activities that change tidal flows [42] and climate change, which is expected to increase wave stress, and the frequency and intensity of storms [43].

Studies on the effects of OA on seagrasses so far have mainly focused on how elevated CO_2_ concentrations will affect the productivity, light requirements and nutrient content of seagrasses [44–47]. However, the net impact of higher CO_2_ is still unclear because there are many ambient variables that could ameliorate or aggravate the effects of CO_2_ increase. The interactions between an increase in CO_2_ and hydrodynamic conditions have not been addressed in any depth, in spite of the importance of the latter at different levels in seagrass ecosystems [48–51]. Likewise, little attention has been given to the effect of hydrodynamics on DOC flux in seagrass meadows. Recent studies have highlighted the direct relationship between ecosystem productivity and DOC flux [28]. Therefore, if hydrodynamic conditions affect photosynthesis, and thus productivity [40], current velocity may also have a significant effect on the DOC flux released by seagrass populations, which in turn could affect the flux of C in coastal communities. Therefore, our aim in this study is to explore the interactive effects of ocean acidification and water current velocity on productivity and the release of DOC in the temperate seagrass *Zostera noltei*.

## Material and methods

### Experimental design

**Fig 1:**
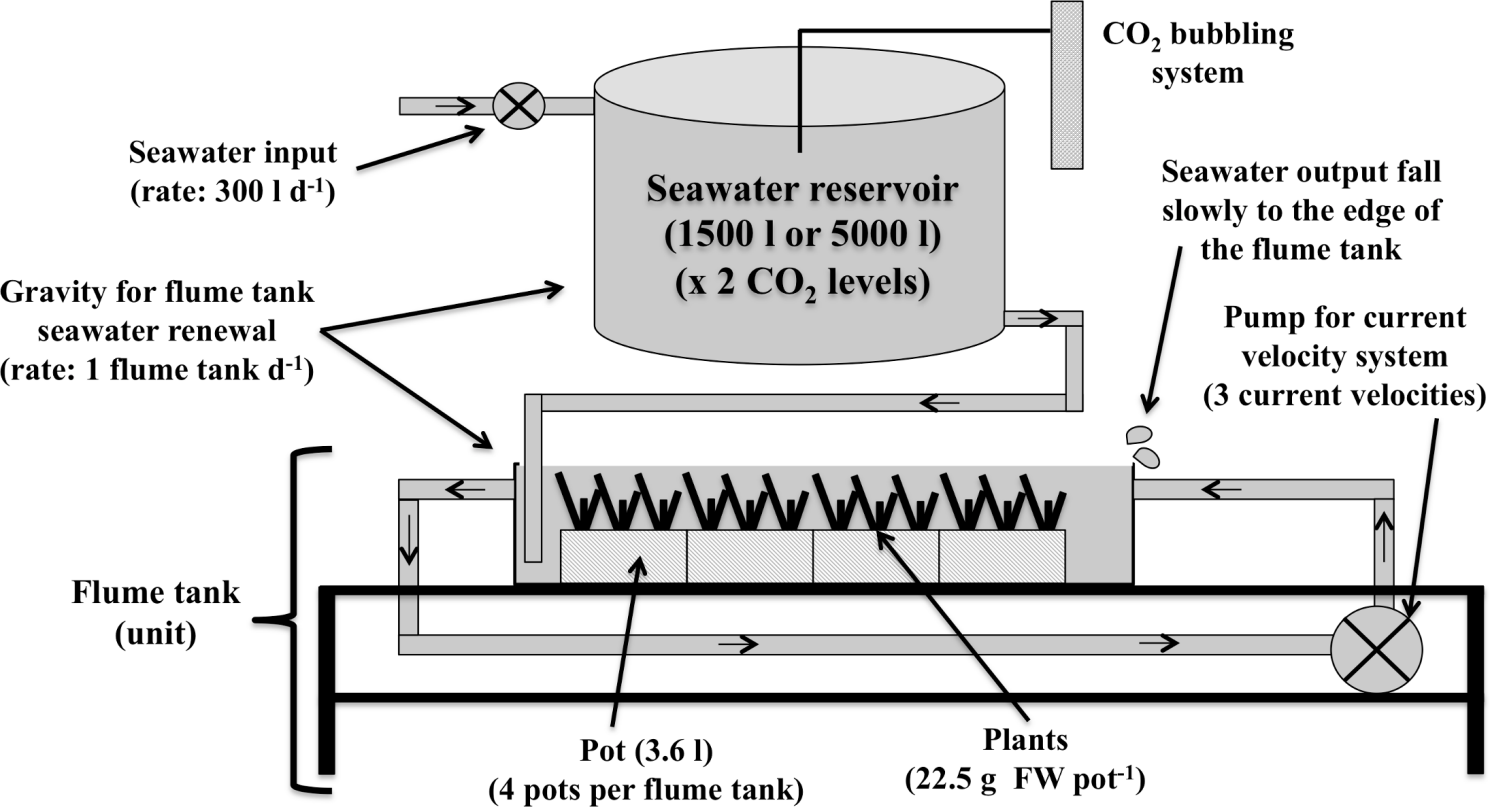
Simplified diagram of the open-water mesocosm system.

This study was conducted on the temperate seagrass *Zostera noltei* Hornemann in an open-water outdoor mesocosm system at the Royal Netherlands Institute for Sea Research (NIOZ), the Netherlands, during a four-week period in the summer of 2014. This time span is long enough to detect any treatment-driven changes in physiological and morphological traits in this fast-growing species (e.g. [52]). Twelve small flume tanks were used to expose plants to three contrasting current velocities (i.e., four flume tanks per current velocity), which were connected to two large seawater reservoirs (volume ca. 1,500 l and 5,000 l). The twelve flume tanks were constructed as independent rectangular stainless-steel containers (13 × 26 × 130 cm). Each flume tank used individual water pumps (further details are given in Peralta et al [53]). The reservoirs fed the flume tanks with seawater set at two pH levels (i.e. six flume tanks per pH level). The factors were manipulated in a fully crossed design, resulting in a total of six treatment conditions with two replicates each (12 units). Each reservoir was filled daily with ca. 300 l of pre-filtered (filter size = 2 μm) water from the Oosterschelde estuary with the aid of a pump. The reservoirs were placed higher than the flume tanks so that gravity could be used to fill them with treated seawater at a turnover time of 1 d. Excess seawater slowly flowed out over the edge of the flume tanks (Fig 1). This high rate of seawater renewal ensured the maintenance of homogenous temperature among the flume tanks. Four stainless-steel pots (12 × 12 × 25 cm) were allocated to each small flume. Pots were completely filled (3.6 l) with a homogeneous mix of clay, sand and gravel. In each pot, about 150–170 individuals of *Z*. *noltei* shoots were planted individually by hand (ca. 22.5 ± 0.5 g FW pot^-1^), resulting in a total of ca. 8000 shoots planted among all units (the twelve flume tanks where levels of the factors CO_2_ and velocity were applied). Ambient light and temperature conditions were used for the duration of the experiment (ca. 1,250 mol photons m^–2^ d^–1^ and 23.5°C measured daily at 10:00 am). To reduce differences in nutrient availability in the water column resulting from the effects of current velocity on sediment flux, all sediment was thoroughly washed and the water column was renewed daily in each flume tank. The total fresh plant biomass of each pot was measured both at the start and end of the experiment. Algae, epiphytes and dead leaves were removed daily, and leaves were weighed for fresh biomass.

### Field plant collection

Individual shoots of *Z*. *noltei* were randomly collected from a bed in the intertidal zone from the mudflats of Viane (51°39N, 4°01E; Oosterschelde estuary), located in the southwestern part of the Netherlands. The permits for collecting seagrass were issued by the province of Zeeland (local government) under the numbers 09002713/NB.08.068 and 12076427. Healthy-looking shoots with intact rhizomes were transported to the laboratory within 60 min of collection in an ice chest, where they were cleaned and visible epiphytes were removed. Before the start of the experiment, plants were kept in aerated natural water for five days under sub-saturating light conditions (ca. 150 μmol photons m^-2^ s^-1^) at 20°C in a 16:8 h light:dark cycle.

### Acidification and water current velocity

Acidification was manipulated in the two large reservoirs with one (1,500 l) maintained for current pH (CpH) and another bigger one (5,000 l) maintained for forecasted pH (FpH). Water properties (temperature, salinity and pH) were measured daily in both reservoirs (at 9:30, 13:00 and 19:00 local hour) to detect any time-dependent effect (Table 1). The CpH reservoir was kept under control conditions (mean target pH along total scale pH = 8.10). The FpH reservoir was manipulated according to the scenario forecasted by the Intergovernmental Panel on Climate Change (IPCC 2013) and was acidified via bubbling with a CO_2_ enriched gas mixture (Westfalen Gassen Netherland BV 250 bar) (mean target pH along total scale pH = 7.65). The FpH reservoir was spiked only once per day, just before filling the reservoir during high tide. Changes in CO_2_ concentration were controlled through daily measurements of water pH, salinity and temperature (at 9:30, 13:00 and 19:00 local hour) at the top of the seagrass canopy in each flume tank. Electrodes were calibrated weekly using standardized TRIS buffers. As a consequence of plant productivity, the pH in each flume tank varied slightly throughout the day (range from 7.8 to 7.9 in FpH, and from 8.1 to 8.3 in CpH) but the high rate of water renewal maintained pH at an almost constant level during the whole day. Seawater samples were collected weekly, immediately filtered (Whatman GF/F filters, 0.7 μm) and stored before adding HgCl_2_ at 4°C. Total alkalinity (TA) was measured by Gran titration using a 888 pH electrode Metrohm meter combined with a glass electrode (Metrohm 6.0259.100), and calculated by the nonlinear least-square method. Carbon chemistry parameters were derived using pH (on the total scale), total alkalinity (TA), temperature and the CO2SYS package [54] with the K1 and K2 constants from Mehrbach et al [55], as modified by Dickson & Millero [56], and the KHSO_4_ constant from Dickson [57]. The pH, TA, temperature, salinity and carbon speciation measured in each flume tank are shown in Table 2. Current velocity was manipulated in the small flume tanks. The low (LV), medium (MV) and high velocities (HV) were set to approximately 0.01, 0.10 and 0.35 m s^-1^, respectively, using individual water pumps for each flume. The selected velocities are within the range of the lowest and highest velocities found at vegetated intertidal areas in the Oosterschelde estuary [53,58].

**Table 1:** Daily water properties in the two large reservoirs at different times across the experimental period.

Reservoir	Time	pH		Temperature	
		Mean ± SE	*p-value*	Mean ± SE	*p-value*
CpH reservoir	9:30	8.12 ± 0.05	0.314	23.3 ± 0.7	0.714
	13:00	8.12 ± 0.01		23.4 ± 0.5	
	19:00	8.14 ± 0.03		23.6 ± 0.7	
FpH reservoir	9:30	7.66 ± 0.03	0.457	23.3 ± 0.2	0.748
	13:00	7.67 ± 0.05		23.4 ± 0.2	
	19:00	7.6 ± 0.04		23.5 ± 0.3	

Data are means ± SE, n = 30. Salinity ~ 30 ppt. *p-value* derived from the one-way ANOVA analysis. CpH = Current pH, FpH = Forecasted pH.

**Table 2:** Summary of seawater chemistry in the different treatments.

Factors		TA (μmol kg^-1^)	pH_T_	DIC (μmol kg^-1^)	pCO_2_ (ppm)
pH	Velocity				
CpH	LV	2,530 ± 18	8.1 ± 0.02	2,232 ± 14	372 ± 15
FpH	LV	2,457 ± 6	7.8 ± 0.02	2,314 ± 11	820 ± 34
CpH	MV	2,642 ± 71	8.1 ± 0.02	2,363 ± 57	448 ± 13
FpH	MV	2,484 ± 11	7.8 ± 0.02	2,340 ± 13	835 ± 55
CpH	HV	2,471 ± 8	8.1 ± 0.02	2,214 ± 9	436 ± 27
FpH	HV	2,473 ± 11	7.8 ± 0.01	2,328 ± 8	810 ± 26

Data are means ± SE, n = 8. Salinity ~ 30 ppt and temperature ~ 20.9°C. TA = Total alkalinity, pH_T_ = total pH, DIC = dissolved inorganic carbon, pCO_2_. LV = Low velocity, MV = Medium velocity, HV = High velocity, CpH = Current pH, FpH = Forecasted pH.

### Sample collection

The DOC flux (i.e. the rate of change in DOC concentration between final and initial sample) was determined weekly for each small flume tank. A positive DOC flux denotes a system that tends to release DOC (DOC producer). In contrast, a negative DOC flux denotes a system that tends to take DOC from other systems (DOC consumer). Three water samples per flume tank were taken from the surface using a 50 ml (polyethylene) acid-washed syringe at 3 different times: i) just before sunset, ii) right after the following sunrise, and iii) 6 h after sunrise. Thus, night and light periods for DOC flux were distinguished [23]. To transform the flume tanks into a closed system during the sampling period for DOC, water renewal was halted. To measure the DOC exchange between the flume tank and the atmosphere, samples were collected at the beginning of the experiment (i.e. when flume tanks were filled and before plants were planted) to subtract the atmosphere-water DOC exchange effect. The seagrass biomass planted in the units ranged between 1 to 2.5 g l^-1^, which is within the range of biomass-volume ratios used in previous studies using incubation chambers (e.g. [23]).

The carbon metabolism of *Z*. *noltei* population was assessed using four benthic chambers (ca. 0.7 l) (i.e. incubations), which were haphazardly allotted to each small flume tank at the end of the experiment in (one incubation per pot). Therefore, we used four replicates per unit. The incubations consisted of a rigid polyvinyl chloride cylinder of 8 cm in diameter firmly inserted, using a sharpened side, to a depth of about 5 cm into the sediment and a gas-tight polyethylene plastic bag fitted to each cylinder [59] enclosing *Z*. *noltei* plants. Each incubation had a sampling port to withdraw water samples. To avoid water stagnation inside the incubations, their walls were made flexible so that they could move to some extent by the wind effect. The incubations were installed in the experimental pots in the evening, two hours before nightfall, to avoid collection of resuspended material from the incubations. The first sampling event began 1h after set-up. The enclosed water was sampled through the sampling port using a 50 ml (polyethylene) acid-washed syringe during the same time as the three aforementioned DOC sampling periods. This is the usual time lapse used for this methodology (e.g. [23]) because it is long enough to determine changes in dissolved oxygen (DO) while avoiding oxygen oversaturation in the incubations. To calculate the volume, 20 ml of a 0.1 M uranine solution (sodium fluorescein, C_20_H_10_Na_2_O_5_) was injected into each incubation at the end of the incubation period, allowing 5 min for mixing, and determining the resulting dilution according to Morris et al [60]. The average water volume enclosed in the incubations was 0.68 ± 0.02 L. Once the polyethylene plastic bag was removed, the *Z*. *noltei* biomass was collected (including belowground biomass), rinsed, dried at 60°C and weighed.

Biomass samples for carbohydrates (in both aboveground and belowground tissues) were collected at the end of the experiment. Plant production was estimated through differences in biomass measured in each pot at the beginning and end of the experiment.

### Laboratory analysis

DOC flux was estimated by changes in DOC during the night and light periods. Water samples from the incubations were filtered through pre-combusted (450°C for 4 h) GF/F filters and kept frozen in acid-washed material (glass vials encapsulated with silicone-teflon caps) until analysis. Concentrations of DOC were derived by catalytic oxidation at high temperature (720°C) and NDIR by using a Shimadzu TOC-VCPH analyzer. DOC-certified reference material (Low and Deep), provided by D. A. Hansell and W. Chen (University of Miami; http://yyy.rsmas.miami.edu/groups/biogeochem/CRM.html), were used to assess the accuracy of the estimations. Net DOC flux was calculated as the difference between the final and the initial DOC concentrations in the water samples. Then, the DOC flux was calculated using the following formula:

$$DOC\;flux=\frac{{DOC}_{f}(\frac{mgC}{l})-{DOC}_{0}(\frac{mgC}{l})}{T_{f}(h)-T_{0}(h)}*\frac{V_{ft}\;(l)}{{PB}_{ft}(gFW)}*\frac{1000\;\mu molC}{12\;mgC}$$

where DOC_f_ and DOC_0_ are the DOC concentrations at final (T_f_) and initial (T_0_) time, V_ft_ is the volume of the flume tank and PB_ft_ is the plant biomass in the flume tank.

Daily rates of DOC flux were calculated by the sum of the hourly DOC flux in light multiplied by photoperiod and the hourly DOC flux in night multiplied by night hours. Thus, when net DOC flux was positive, the system behaved as a net DOC producer. In contrast, when net DOC flux was negative, the system behaved as a net DOC consumer.

Samples of water for measuring DO concentration were fixed immediately after collection, kept in darkness and refrigerated (4°C), and DO was determined using a spectrophotometric modification of the Winkler titration method [61,62]. Changes in DO concentration between the three different collection periods (S1, S2 and S3) were used later to estimate the net primary production (NPP), gross primary production (GPP) and respiration (R). The R during the night hours was estimated as the difference in O_2_ concentrations between sampling periods S2 and S1. The NPP during light hours was estimated as the difference in O_2_ concentrations between sampling periods S3 and S2. GPP during light hours was calculated as the sum of hourly rates of R and NPP, assuming similar night and light respiration. Metabolic rates were converted into carbon units assuming photosynthetic and respiratory quotients of 1, a value that is widely used in seagrasses (e.g. [63]). Daily rates of GPP were calculated by multiplying the hourly GPP by photoperiod. Daily rates of R were calculated by multiplying the hourly R by 24 h, assuming that R is the same during night and light hours. Finally, daily rates of NPP were estimated as the difference between daily rates of GPP and R.

The concentration of non-structural carbohydrates (sucrose and starch) was measured in duplicate leaf and rhizome samples from each incubation. Samples were freeze-dried and ground prior to analysis. Total non-structural carbohydrates were measured following Brun et al [64]. Sugars (sucrose and hexoses) were first solubilized by four sequential extractions in 96% (v/v) ethanol at 80°C for 15 min. The ethanol extracts were evaporated under a stream of air at 40°C and the residues were then dissolved in 10 ml of deionized water for analysis. Starch was extracted from the ethanol-insoluble residue by soaking it in 1 N NaOH for 24 h. The sucrose and starch content of the extracts was determined spectrophotometrically using resorcinol and anthrone assays with an absorbance of 486 and 640 nm, respectively, and using sucrose as the standard.

### Data and statistical analysis

Prior to any statistical analysis, data were checked for normality (Shapiro-Wilk normality test) and homocedasticity (Bartlett test of homogeneity of variances test). Statistical differences in daily water properties at different times in each large reservoir were analysed using a one-way ANOVA. Statistical differences between factors (pH and current velocity) were analysed using a 2-way ANOVA. When significant differences were found, a Tukey post-hoc test was applied. After several transformations, values of R did not meet the normality assumption; therefore, differences in R among treatments were analysed using the Kruskal-Wallis test with the Wilcoxon signed-rank test. Data are presented as mean ± SE. The significance level (α) in all tests was set at a probability of 0.05. Statistical analyses were computed with *R* 3.0.2 (*R* Development Core Team 2013).

## Results

### Abiotic variables

Mean daily water temperature showed an unimodal response, averaging 23.4 ± 0.8°C and ranging from ca. 21°C (midnight) to ca. 25°C (midday) in all experimental units. This low variation in daily temperature was due to the high rate of water renewal (see Methods section). Regarding light conditions, mean daily light level at the water surface at 10:00 am was about 1,250 μmol photons m^–2^ s^–1^ in all experimental units.

### Effects on seagrass metabolism

**Fig 2:**
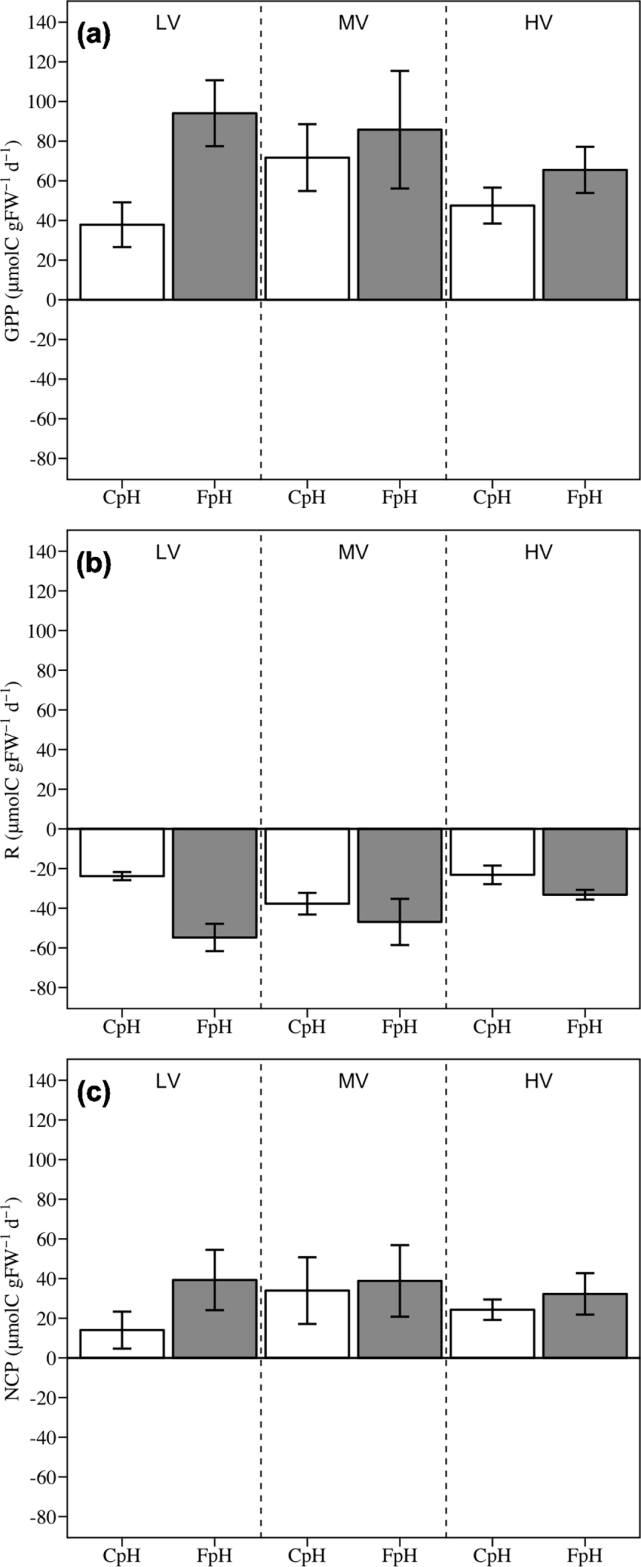
**Effects of seawater acidification and current velocity on (A) gross primary production (GPP), (B) respiration (R) and (C) net primary production (NPP).** LV = Low velocity, MV = Medium velocity, HV = High velocity, CpH = Current pH, FpH = Forecasted pH.

Lowering the pH led to significant responses in GPP and R, with values under FpH about 1.5 times higher than those under CpH for both GPP and R (Fig 2A & 2B). However, lowering the pH did not lead to significant differences in NPP (Fig 2C). Overall, changes in current velocity did not result in significant responses for GPP, R and NPP. Nevertheless, values found under medium velocity and CpH conditions were around 1.6, 1.7 and 1.8 times higher for GPP, R and NPP, respectively, than the means of the other two levels of water velocity under CpH conditions. Under more acidic FpH conditions, the differences between current velocity levels almost disappeared. The interaction between pH and current velocity resulted in significant differences in R, with the treatment combination of FpH + LV showing higher R than the treatment combination of CpH + HV (Fig 2 and Table 3).

**Table 3:** Two-way ANOVA results for the effect of seawater acidification and current velocity (CV) on gross primary production (GPP), respiration (R), net primary production (NPP), dissolved organic carbon flux (DOC) and biomass loss (BL).

	GPP	R	NPP	DOC	BL
pH	**0.046**	<**0.01**	0.255	0.159	0.252
CV	0.343	0.212	0.669	<**0.001**	**0.005**
pH:CV	0.374	**0.014**	0.705	0.664	0.199

Bold numbers indicate significant differences at *p* < 0.05.

### Effects on non-structural carbohydrate content

**Fig 3:**
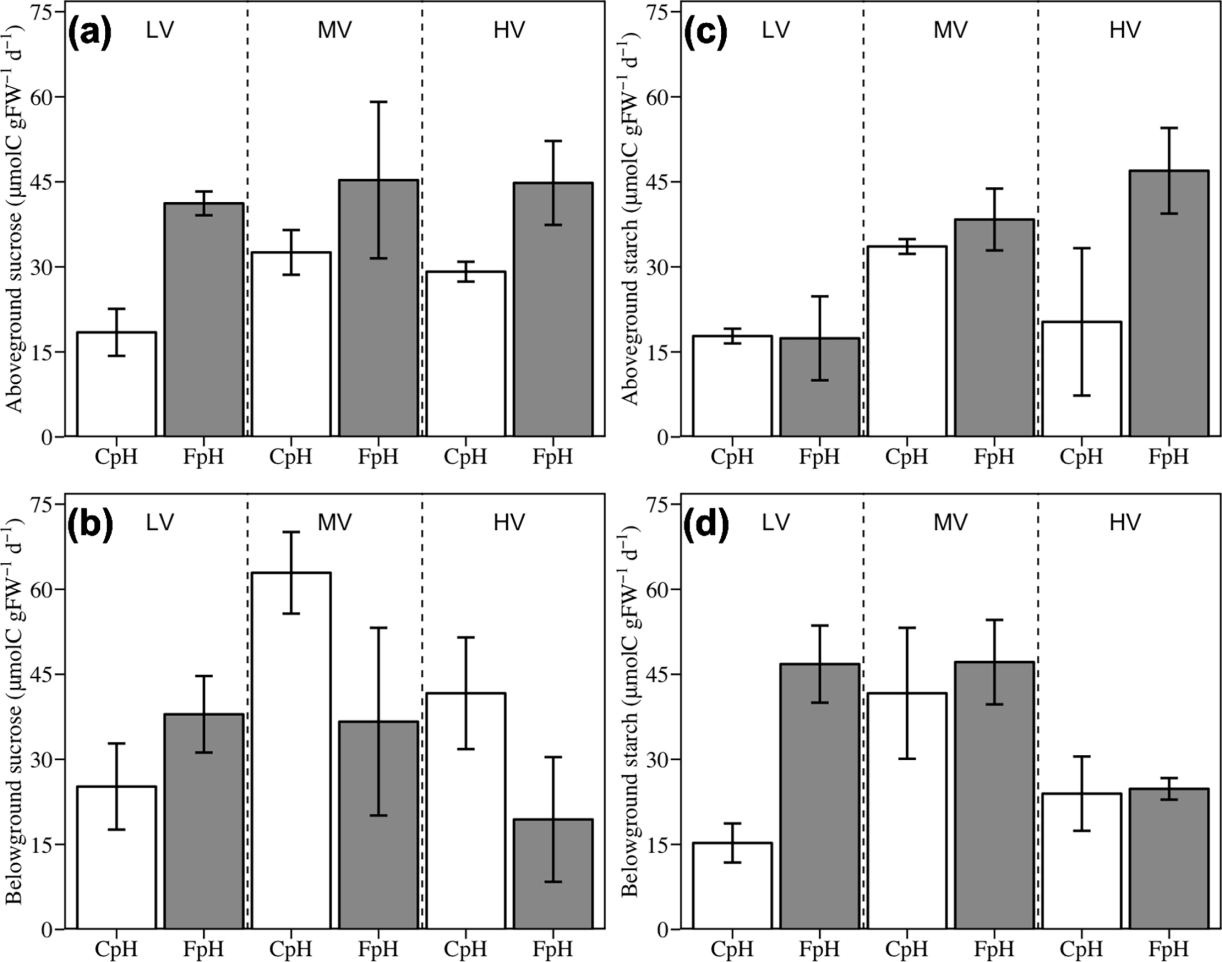
**Effects of seawater acidification and current velocity on (A) aboveground sucrose, (B) belowground sucrose, (C) aboveground starch and (D) belowground starch.** LV = Low velocity, MV = Medium velocity, HV = High Velocity, CpH = Current pH, FpH = Forecasted pH.

Sucrose levels of aboveground tissues were significantly higher under lower pH, with seagrasses subjected to FpH averaging around 1.6 times the value of those subject to CpH (Fig 3A and Table 4). In contrast, no significant effects of pH were found on sucrose content in belowground tissues (Fig 3B and Table 4), nor were they found on starch content in both above and belowground tissues (Fig 3C and 3D and Table 4). Current velocity did not result in any significant responses in either sucrose or starch content. Interactions between pH and current velocity did not result in any significant response in carbohydrate content; however, the sucrose content of the belowground tissues was around 0.4 times lower in the treatment combination of LV + CpH than the mean of all other treatment combinations (Fig 3C and 3D and Table 4).

**Table 4:** Two-way ANOVA results for the effect of seawater acidification and current velocity (CV) on aboveground sucrose, belowground sucrose, aboveground starch and belowground starch (mg sucrose or starch g DW^-1^).

	Above sucrose	Below sucrose	Above starch	Below starch
pH	**0.0232**	0.210	0.131	0.060
CV	0.433	0.195	0.085	0.071
pH:CV	0.766	0.200	0.220	0.139

Bold numbers indicate significant differences at *p* < 0.05.

### DOC flux

**Fig 4:**
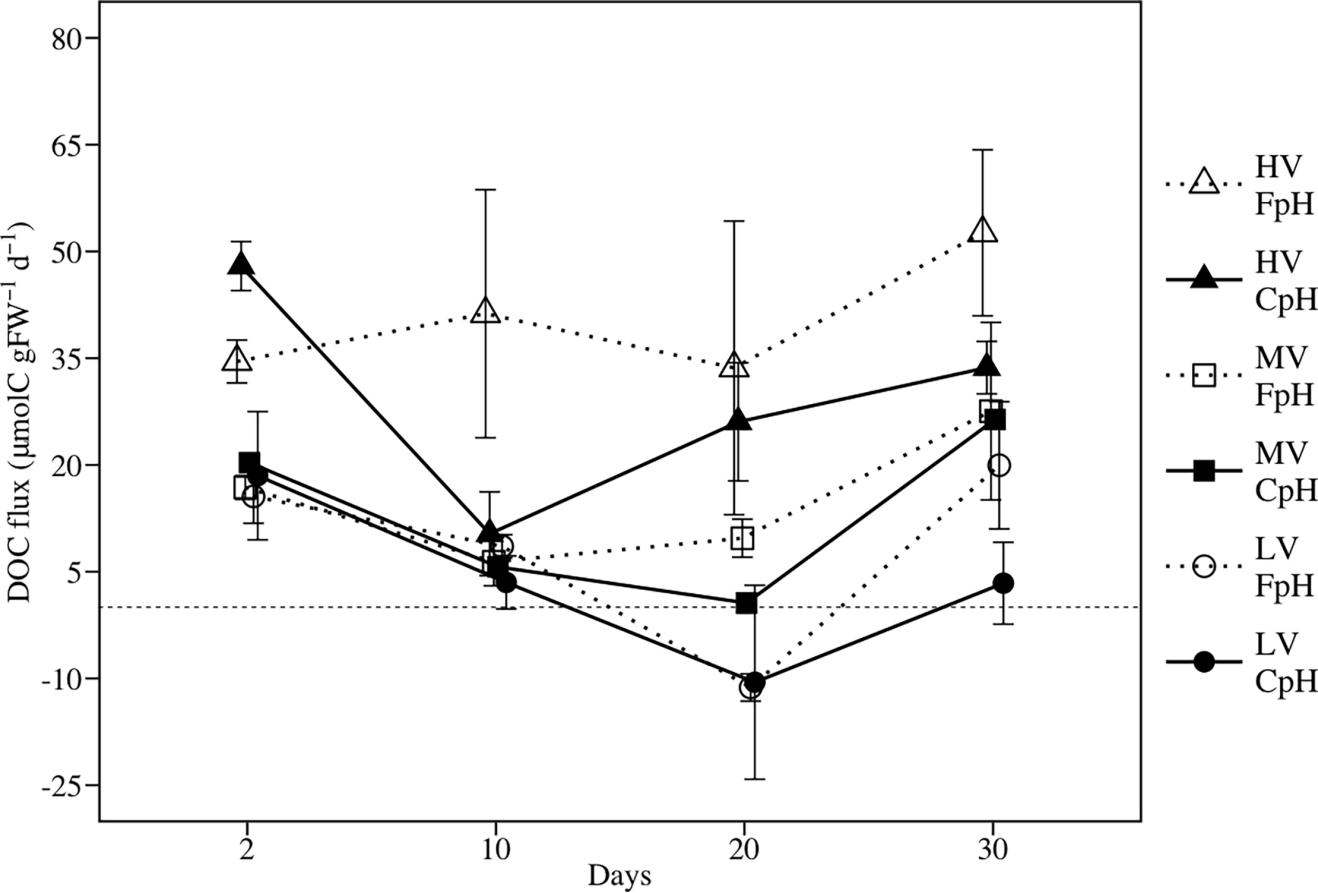
Effects of seawater acidification and current velocity on net DOC flux. LV = Low velocity, MV = Medium velocity, HV = High velocity, CpH = Current pH, FpH = Forecasted pH.

Lower pH did not have a significant effect on DOC flux, whereas current velocity significantly affected DOC flux (Fig 4 and Table 3). DOC flux under HV was about 6 times higher than flux under LV. Also, DOC flux under MV was around 2.4 times higher than flux under LV. Overall, DOC flux varied from around 16% of NCP in LV to 64% of NCP in MV to even exceeding the NCP under HV (120% of NCP). There was a non significant interaction between pH and current velocity on DOC flux.

### Biomass loss

Lower pH did not result in a significant response in biomass loss. In contrast, current velocity produced significant differences in this variable, with biomass loss being higher under LV current (29 ± 3.1%) than under MV (21 ± 2.6%) and HV (16 ± 2.5%) currents. The interaction between pH and current velocity (Table 3) did not result in overall significant differences. However, the HV and FpH combination resulted in the lowest levels of biomass loss, and was 1.7 times lower than the mean of all other combinations together (13.7% vs. 23.5% biomass loss).

## Discussion

The two factors assayed (pH and current velocity) produced significant changes in the carbon metabolism and dissolved organic carbon (DOC) flux in *Z*. *noltei*. Lower pH mainly enhanced seagrass productivity, whereas current velocity mainly enhanced DOC flux. Throughout the experiment, *Z*. *noltei* remained net autotrophic, independently of the treatment condition, and maintained high productivity under all the conditions tested. On average, GPP was 65 ± 7 μmol C gFW^-1^ d^-1^, which is within the range of values described by Duarte et al [29] for a large number of seagrass species. A significant increase (150%) in GPP and R under the more acidic forecasted pH conditions was found. This may indicate that photosynthesis of *Z*. *noltei* is carbon limited at the current inorganic carbon concentration of seawater (i.e. CO_2_), as found in previous studies on this species (e.g. [65,66]), and also in studies on other seagrass species [44,45,67]. Hence, *Z*. *noltei* may benefit from future CO_2_ enrichment through the enhancement of photosynthetic rates at higher CO_2_ concentrations [65]. These higher CO_2_ concentrations may lead to significant higher uptake of C, which could in turn be stored in the tissues of *Z*. *noltei* (e.g. increase in non-structural carbohydrates reserves as found in this study) or exported in particulate or dissolved form (as also found in this experiment), contributing to an increase in the productivity of adjacent communities.

We acknowledge that the presented experimental design, although have been widely used in the literature for ocean acidification research (see Cornwall & Hurd 2016 [68]), can be considered inappropriate according to Humbert 1984 [69]. The six treatments used here were independent respect the factor current velocity (i.e. each unit had their own flume tank and water pump) but inter-dependent respect the factor pH, as all units of each pH conditions were feed with the same large reservoir (i.e. one header tank of seawater for CpH and another for FpH where the CO_2_ was manipulated). Then, a single chance of motor failure (e.g. water contamination event or other kind of intrusion) could have produced a “spurious treatment effect”. Experimental logistics prevented the use of a pH header tank for each unit. However, the use of two large reservoirs that feed all replicates of each pH treatment aided to reduce experimental variability and to maintain the required water conditions for a longer time (i.e. water temperature, alkalinity, etc.), as demonstrated by our daily monitoring of water physical-chemical variables (see results section, abiotic variable subsection and Table 2). In addition, each unit of each pH treatment was manipulated as an independent system (i.e. with their own independent maintenance system, their own water pump etc.) and all experimental units in one pH treatment was equally exposed to the same conditions as all experimental units in the another pH treatment, which reduced the possible bias according to Hurlbert 2013 [70]. On the other hand, the rate of water input into the two large reservoirs and in each units were very high (~300 l d^-1^ for large reservoirs and ~50 l d^-1^ for units). Hence, microorganisms in water do not biologically modify seawater carbonate chemistry significantly over the duration of the experiment as it noted in Table 1. Therefore, the possibility of “spurious treatment effect” as a consequence of experimental design, if occurred, should have been very low and then, the variability associated with this experimental design does not mask the main results of the research.

**Fig 5:**
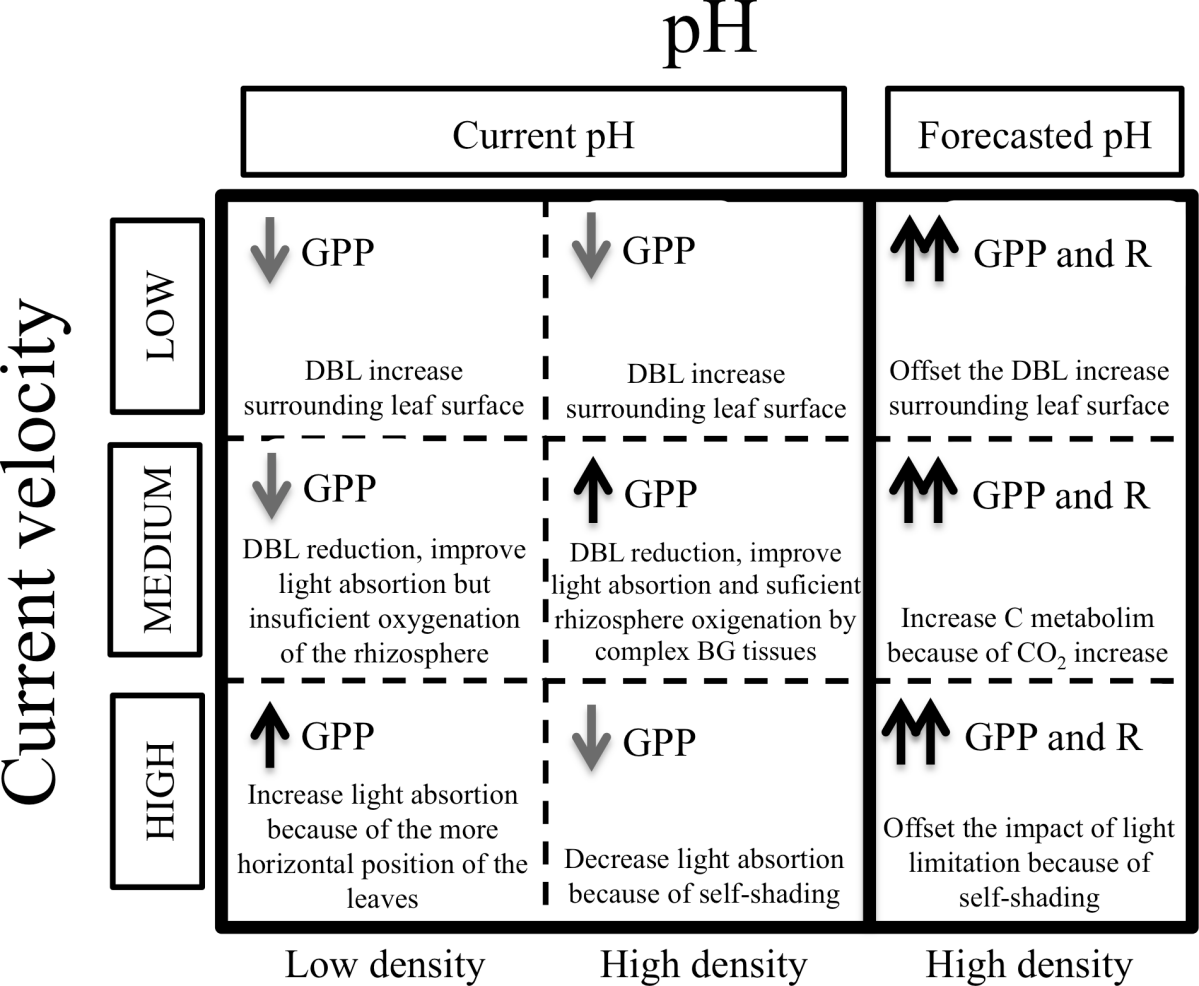
Conceptual model showing the likely effects of CO_2_ concentration, current velocity and shoot density on gross production (GPP) and respiration (R). (↑) Increase, (↓) Decrease, (↑↑) Significant increase. Current pH means the actual sea pH (pH = 8.1). Forecasted pH means the ocean acidification scenario forecasted by the Intergovernmental Panel on Climate Change (pH = 7.65).

Current velocity did not have significant effects on plant productivity, although plants subject to MV under CpH conditions had higher GPP (170%) than the average of the other two velocity levels. Under low current velocities, the thickness of the DBL surrounding the leaf surface likely increases, which reduces carbon uptake, as previously demonstrated for inorganic carbon [35–38] and other nutrients [48,71,72]. In contrast, under high current velocities, self-shading in the population increases since leaves orient themselves almost horizontally in the strong current, collapsing onto each other and affecting light absorption and thus productivity [40]. The intermediate current velocity likely allows for a more favourable balance between DBL reduction and light absorption, leading to an increase in plant productivity. Some previous studies conducted in the same small flumes have shown the opposite trends (mainly at high current velocity) to those found in this experiment [51,53,72]. For example, in these previous studies, higher current velocities actually favoured the growth and development of *Z*. *noltei* plants, mainly because of the more horizontal position of their leaves, which enhanced light capture. The relatively lower plant growth under MV was explained by insufficient oxygenation of the rhizosphere, which requires energy to offset the effects of the anoxic sediment [73–75]. However, the main difference between our experimental design and that of these previous studies is shoot density. We used a high shoot density (c.a. 5,650 shoots m^-2^) in our design, whereas shoot density was one order of magnitude lower (c.a. 150 shoots m^-2^) in these previous studies. Under such experimental conditions, the interaction between density and current velocity can produce opposite effects. Under higher shoot density and MV conditions, the associated higher complexity of the belowground network in *Z*. *noltei* beds may lead to greater oxygenation of the rhizosphere; under HV conditions, light capture may decrease because of increased self-shading by leaves in the canopy. The interactions between pH, current velocity and shoot density deserve further research. However, the differences in GPP between different levels of current velocity disappeared under FpH, which suggests that *Z*. *noltei* plants were CO_2_ saturated under more acidic conditions. Thus, CO_2_ enrichment may limit the impact of light limitation on seagrasses [76] by offsetting the lower light absorption under HV conditions, while also probably offsetting the increase in DBL thickness surrounding the leaf surface under LV conditions. Thus, the interactive effects of pH and current velocity on the productivity of *Z*. *noltei* populations are not as straightforward as expected: under current levels of pH, current velocity may have a positive effect depending on shoot density; under the lower pH levels forecast for the future, this positive effect of current velocity may disappear (Fig 5).

Regarding the effects on non-structural carbohydrates, sucrose increased in aboveground tissues under FpH conditions, while a non-significant response was found in belowground sucrose and in both above- and belowground starch content. However, belowground starch increased by 30–260% in plants subjected to the treatment conditions of LV + CpH (Fig 3 and Table 4). This is consistent with previous short-term experiments on the effects of OA in seagrasses, which showed an increase in the concentration of non-structural belowground carbohydrates [47,77]. Carbon reserves are essential for seagrass survival under stressful environmental conditions because environmental stressors, such as periods of light limitation [78–81], ammonium toxicity and/or sediment anoxia [64,82], increase plant demand for carbon. Under these stressful conditions, plants are forced to use stored carbohydrates to maintain their carbon skeletons and provide energy during these periods of low C fixation through photosynthesis.

**Fig 6:**
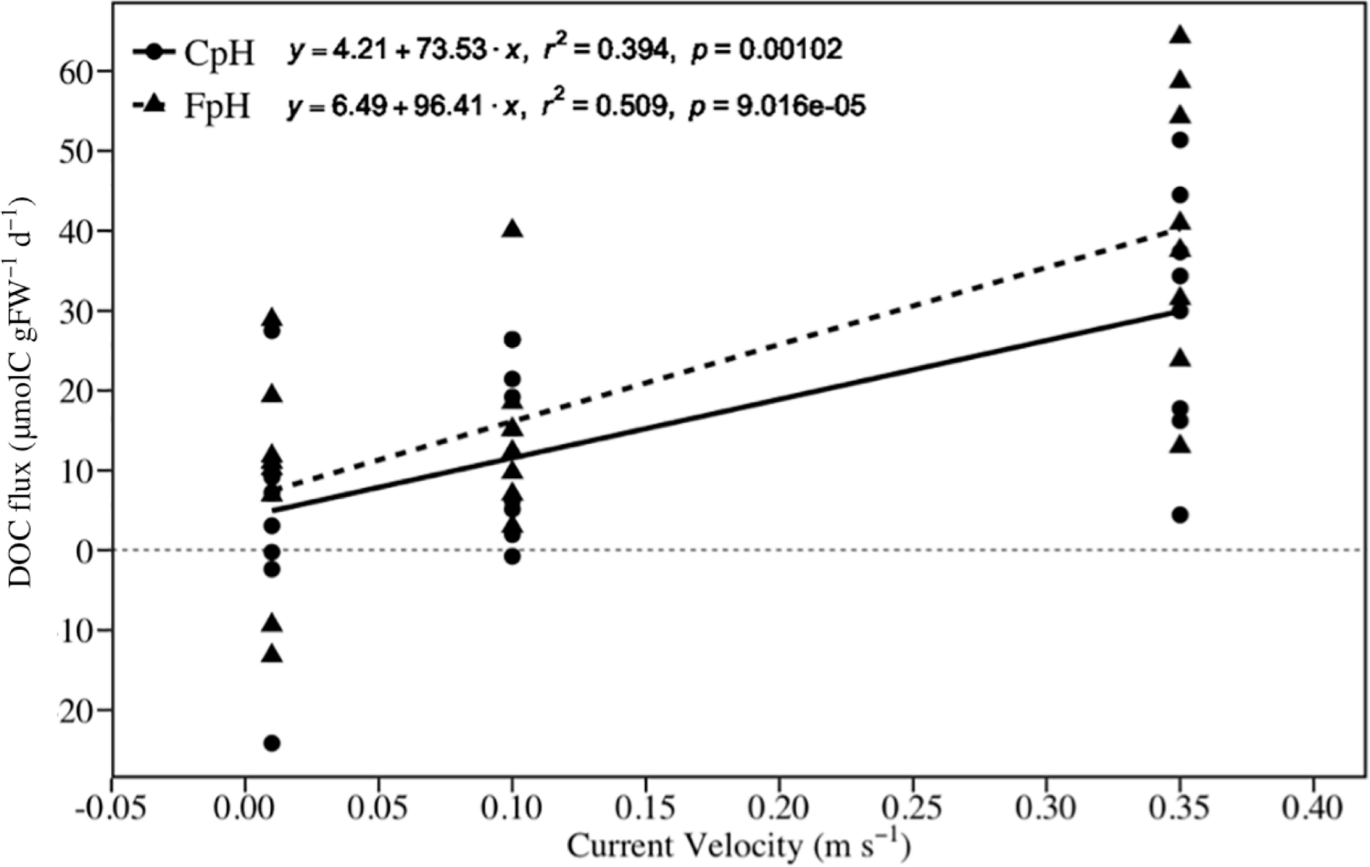
Relationship between current velocity and DOC flux. CpH = Current pH, FpH = Forecasted pH.

One of the most noteworthy results of this study was the positive relation between DOC flux and current velocity, independently of pH level. It is known that the DOC released to the ocean depends on many factors (e.g. temperature, light, community respiration, nutrients, etc. [25, 28]). The present study demonstrates that among these factors, hydrodynamic conditions is an important factor determining the dynamic of DOC in seagrass communities (Figs 4 and 6 and Table 3). Our results show that circa of 50% of the variability in DOC release in our experimental seagrass community was explained by hydrodynamic conditions (Fig 6). The remainder may be explained by the existence of other factors that are also known to affect the release of DOC in seagrass communities such as temperature, light, nutrients, bacterial use of DOC, etc. The positive relation between DOC flux and current velocity can be attributed to the sum of two different processes. First, as demonstrated by this work, increasing current velocity results in higher GPP of *Z*. *noltei* population, and since a part of the GPP is released as DOC [28], increasing current velocity indirectly results in a higher release of DOC. Second, seagrass ecosystems store large amounts of particulate organic carbon in the sediment [83], which can potentially be transformed into DOC through the activity of heterotrophic bacteria [84,85]. Although the relative contribution of each compartment (e.g. DOC released from sediment) to the net DOC flux in seagrass communities remains poorly understood [28], higher current velocity is expected to also enhance the transfer of DOC from the sediment to the water column, as demonstrated in other compounds [40,86–88]. This finding has interesting ecological implications since the DOC released from seagrass populations means quicker and more efficient transfer of carbon and energy from primary producers to higher trophic levels; thus, seagrass populations thriving in areas with higher hydrodynamics may produce higher DOC fluxes.

Besides seawater acidification and current velocity, other factors linked to climate change, such as warming, may severely impact the productivity and even survival of marine organisms [89]. By the end of this century, mean global surface temperatures are projected to increase by ~3–4°C [90]. Temperature is a key factor for seagrass health, growth and community metabolic rates [10]. Thus, increases in temperature may exacerbate or alleviate the results found here. For example, the Metabolic Theory of Ecology (MTE) predicts that respiration rates will increase more quickly under warming scenarios than primary production rates [91–93], which results in a reduction of the P:R ratio and a greater chance of shifting systems into heterotrophy. On the other hand, recent studies have shown that the net DOC flux in seagrass communities are positively correlated with water temperature [23,28]. Hence, future research should delve into the interactions between ocean acidification and current velocity with other environmental or anthropogenic stressors linked to climate change, such as warming, in order to understand and mitigate the effects of this global threat on seagrass ecosystems.

In summary, the best scenario in this study for *Z*. *noltei* was under OA and high current velocity, which led to a significant increase in productivity and non-structural carbohydrate concentrations, especially in aboveground sucrose and starch, and the lowest levels of biomass loss. In addition, a direct relationship between current velocity and DOC flux was recorded at the end of the experiment under both OA and control conditions. Since both factors (i.e. OA and hydrodynamics) are expected to increase under climate change, and without considering other indirect interactions such as warming, sea level change, herbivory, etc., seawater acidification and higher current velocity may produce a favourable scenario for *Z*. *noltei* populations, increasing their productivity, their non-structural carbohydrate levels and release of DOC, which means a quicker and more efficient transfer of carbon and energy to higher trophic levels, and higher resistance and resilience to external stressors.
